# Real-World Use of ARNI Within GDMT in HFrEF Patients with and Without Atrial Fibrillation: A Retrospective Analysis of Cardiac and Renal Functions and Clinical Outcomes

**DOI:** 10.3390/jcdd12090328

**Published:** 2025-08-26

**Authors:** Niccolò Bonini, Marta Mantovani, Marco Vitolo, Kevin Serafini, Enrico Tartaglia, Francesca Rampini, Francesca Grossule, Benedetta Cherubini, Maria Laura Mastronardi, Paola Trapanese, Jacopo F. Imberti, Davide A. Mei, Giuseppe Boriani

**Affiliations:** 1 Cardiology Division, Department of Biomedical, Metabolic and Neural Sciences, University of Modena and Reggio Emilia, 41123 Modena, Italy; bonini.niccolo93@gmail.com (N.B.); marta.atram.mantovani@gmail.com (M.M.); marco.vitolo@unimore.it (M.V.); kevin.serafini15@gmail.com (K.S.); enricotartaglia412@gmail.com (E.T.); francesca.rampini22@gmail.com (F.R.); francesca.grossule@gmail.com (F.G.); bennycher@gmail.com (B.C.); marialauramastronardi@yahoo.it (M.L.M.); trapanesepaola@gmail.com (P.T.); jacopo.imberti@hotmail.it (J.F.I.); davide.mei93@gmail.com (D.A.M.); 2 Clinical and Experimental Medicine PhD Program, University of Modena and Reggio Emilia, 41123 Modena, Italy

**Keywords:** atrial fibrillation, heart failure with reduced ejection fraction, left ventricular ejection fraction, renal function, guideline-directed medical therapy

## Abstract

The aim of this study was to describe changes in estimated glomerular filtration rate (eGFR), left ventricular ejection fraction (LVEF) and clinical outcomes in a real-world cohort of patients with heart failure with reduced ejection fraction (HFrEF) and atrial fibrillation (AF). A total of 321 patients (67 [58–74] years old, 19.3% females) were included; 134 (41.7%) had AF. AF patients were less frequently prescribed angiotensin receptor–neprilysin inhibitor (ARNi), with no differences concerning sodium–glucose transport protein 2 inhibitors (SGLT2is) and had lower median baseline eGFR values. At 6- and 12-month follow-ups, renal function declined similarly in both groups, with no difference in the proportion of patients experiencing an eGFR decrease of ≥30% from baseline. Regarding cardiac remodeling, patients without AF showed a higher proportion of individuals with an LVEF improvement of ≥10% from baseline, however with no differences between groups in LVEF final recovery. During a median follow-up of 582 (339–1481) days, AF patients showed a higher risk of composite outcome (aHR, 95% CI: 2.12, 1.16–3.86) and of hospitalization for heart failure (hHF) (2.80, 1.44–5.46), without differences in all-cause death. Delta eGFR changes with at least a 30% decline in eGFR were associated with a higher risk of the primary endpoint. Despite lower baseline renal function, AF patients exhibited similar LVEF improvement and renal decline, which emphasizes the importance of guideline-directed medical therapy. AF was associated with a higher risk of adverse events, primarily driven by hHF.

## 1. Introduction

Heart failure with reduced ejection fraction (HFrEF) represents a current challenge for cardiovascular (CV) health, and similarly, atrial fibrillation (AF) is the most prevalent cardiac arrhythmia worldwide [1,2]. The two conditions frequently coexist in up to 40% of patients, exacerbating each other’s adverse events [3]. In this complex clinical setting, optimizing guideline-directed medical therapy (GDMT) for HF patients is crucial in mitigating cardiovascular outcomes [4,5,6]. In particular, angiotensin receptor–neprilysin inhibitor (ARNi) showed no increase in AF incidence and consistent efficacy across AF patients in the PARADIGM-HF trial [7], and sodium–glucose transport protein 2 inhibitors (SGLT2is) proved consistent beneficial effects in patients with AF in most RCTs [8,9,10,11]. However, the real-world interplay and management of HFrEF with AF remain complex, often complicated by polypharmacy and drug tolerability [12]. For instance, AF patients frequently present with chronic kidney disease (CKD), which may limit the full optimization of guideline-directed medical therapy (GDMT) [13], particularly due to concerns about a decline in estimated glomerular filtration rate (eGFR). However, the modest reduction in eGFR during GDMT initiation—especially with ARNIs and SGLT2 inhibitors—should not be a cause of therapeutic inertia, as per their significant nephroprotective effect. Moreover, in patients with HFrEF, GDMT is frequently associated with significant positive left ventricular (LV) remodeling. However, the presence of atrial fibrillation (AF) may technically interfere with an accurate assessment of the remodeling parameters, particularly LV ejection fraction (LVEF), due to the irregular rhythm and lack of atrial contribution. This limitation may partly explain why observational studies often report a lower prevalence of HFimpEF among patients with AF, potentially reinforcing the perception that AF attenuates LVEF improvement and limits the extent of reverse remodeling in HFrEF [14].

The aims of this study are the following: (i) to describe longitudinal changes in eGFR and LVEF in a real-world cohort of HFrEF patients stratified by AF status; (ii) to compare clinical outcomes in HFrEF patients with and without AF.

## 2. Materials and Methods

### 2.1. Study Design and Definitions

This was a single-center, retrospective cohort, which included outpatients with HFrEF who were prescribed an angiotensin receptor–neprilysin inhibitor (ARNi) and/or a sodium–glucose transport protein 2 inhibitor (SGLT2i). The study was conducted at the Cardiology Division of our tertiary care University Hospital, from July 2017 to September 2024. Data collection for the Italian Drug Agency (AIFA) therapeutic plans required for the reimbursement of ARNI and/or SGLT2 inhibitors by the national health system included the following elements: symptomatic HFrEF (NYHA class II–III only), physical examination with blood pressure measurement, LVEF ≤ 35% for the first ARNI prescription and LVEF ≤ 40% for the first SGLT2i prescription, prior use of an ACEi or ARB for at least 1 month, and the assessments of concomitant medications, comorbidities, renal function, serum potassium, and natriuretic peptides.

The study protocol was approved by the local Ethics Committee (approval number: 1343/2020), and the study was performed according to the principles of the Declaration of Helsinki.

We calculated eGFR according to Chronic Kidney Disease Epidemiology Collaboration (CKD-EPI) formula, and an eGFR < 60 mL/min/1.73 m^2^ was used as the cut-off for the definition of CKD.

HFrEF was defined as HF associated with an LVEF ≤ 40%, as per the current guidelines. Clinical AF was defined in accordance with the current guidelines by the ECG.

For the purpose of this analysis, we included patients with available information concerning the AF status and follow-up data.

### 2.2. Follow-Up and Adverse Events

Clinical information and biochemistry parameters were collected and reported by local investigators through patient follow-up visits, which were regularly performed every 6 months to renew the AIFA therapeutic plan required for the continued prescription of ARNi or SGLT2i.

Delta changes in LVEF were calculated as follows: from baseline to the first follow-up visit (6 months), i.e., delta LVEF 6-month; from baseline to the second follow-up visit (12 months), i.e., delta LVEF 12-month; and from baseline to the last available follow-up, i.e., delta LVEF overall.

Patients whose LVEF improved reaching values above 35% were defined as improved LVEF (ImpLVEF).

Similarly, delta changes in eGFR were calculated. Patients who experienced a decline in eGFR ≥ 30% from baseline to the last available follow-up were defined as worsening eGFR (WeGFR).

The primary endpoint was a composite of all-cause death and hospitalization for HF (hHF). The secondary exploratory endpoints were the single components of the primary outcome.

### 2.3. Statistical Analysis

Continuous variables are reported as median and interquartile range [IQR] and were compared using appropriate non-parametric tests, while categorical variables are shown as counts and percentages and were compared using the chi-square test.

The incidence rates (IRs) of the primary and secondary endpoints were calculated as the number of events per 100 person-years with the related 95% confidence interval (CI). Kaplan–Meier survival curves were built to illustrate the differences in the survival rates among patients with or without AF and were statistically tested using log-rank test. Cox regression analysis was used to evaluate the association with the risk of the primary and secondary exploratory endpoints. We used 2 covariates models: Model 1 was adjusted for age, sex, ischemic etiology, LVEF and hypertension; and Model 2 was also adjusted for diabetes and eGFR. The results are reported as adjusted hazard ratio (aHR) and 95% CI. The proportional hazard assumption was checked using the Schoenfeld residuals, with no violation detected.

Moreover, we analyzed the relationship between eGFR changes and primary endpoint risk, considering delta eGFR as a continuous variable, modeled using a restricted cubic spline curve with 3 knots. Similarly, we examined the association between delta eGFR from the 6-month follow-up to the last available follow-up and the primary endpoint. In both models, a delta GFR decline of 30% was used as the reference value.

A two-sided *p* < 0.05 was considered statistically significant. All analyses were performed using R (version 4.3.2).

## 3. Results

### 3.1. Study Population

**Figure 1:**
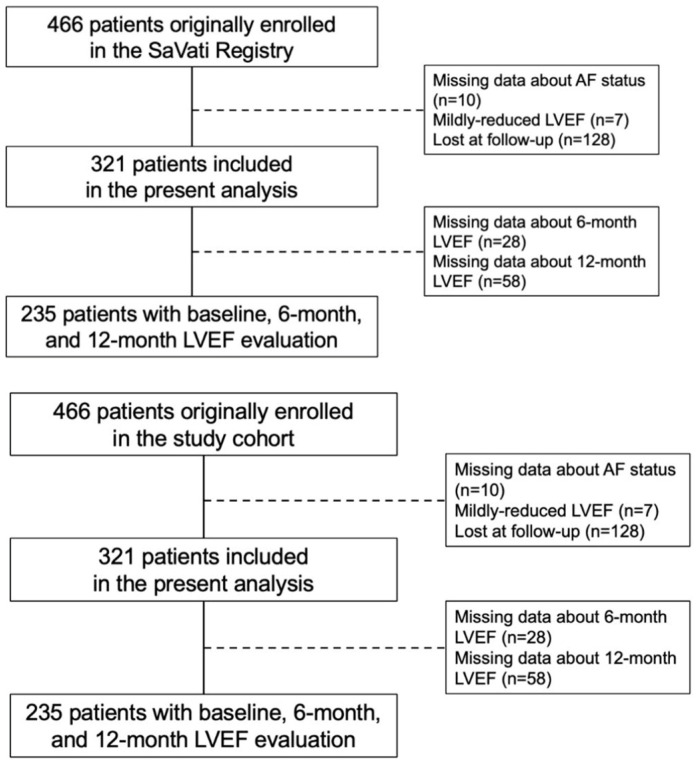
Flow-chart of the study. AF, atrial fibrillation; LVEF, left ventricular ejection fraction.

Of the 466 patients initially enrolled, 321 (median age 67 [58–74] years, 19.3% females) with available AF status and follow-up data were included in this analysis. The flow-chart of the study is reported in Figure 1.

Overall, the cohort predominantly comprised patients in New York Heart Association (NYHA) class 2 [2,3]. Baseline characteristics and treatments of the study population are summarized in Table 1.

The AF patients were older (median age 72 [65–78] years vs. 64 [56–71] years, *p* < 0.001), with a higher prevalence of CKD (37.8% vs. 18.5%, *p* < 0.001) and with a median LVEF of 32% [30–35]. No differences were observed concerning the prevalence of ischemic etiology and other comorbidities between the groups (Table 1). The AF patients had higher median RDW and BPN values compared to the non-AF ones.

Regarding treatments (Table 1), ARNi was prescribed to 75.1% of the patients, more frequently to the non-AF patients (80.2% vs. 67.9%, *p* = 0.017), and no differences were observed concerning the other foundational (“four pillars”) HF therapies between the groups. Antithrombotic therapies were consistent according to the AF status.

### 3.2. Follow-Up Changes in Renal Function and LVEF

The details on LVEF and eGFR variations over the follow-ups (median duration: 582 [339–1481] days) are reported in Table 2.

**Figure 2:**
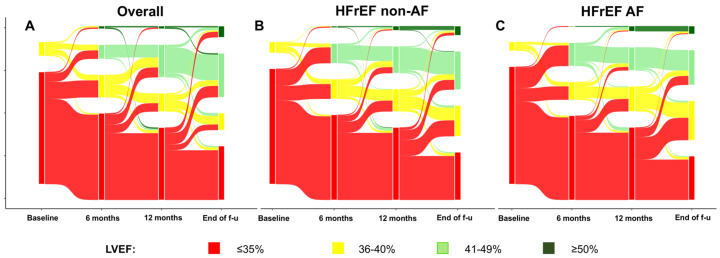
Sankey plot of changes in left ventricular ejection fraction from baseline to median follow-up. Panel (**A**), overall cohort; panel (**B**), non-AF patients; panel (**C**), AF patients. AF, atrial fibrillation; LVEF, left ventricular ejection fraction. The Sankey plots depict the individual changes in LVEF in the overall cohort (panel **A**) and in stratified patients without (panel **B**) and with (panel **C**) AF. f-u: follow up.

Figure 2A–C displays the LVEF changes in the overall cohort and in patients stratified by AF status, showing that the AF patients experienced less reverse remodeling during follow-up. Indeed, LVEF improved from 31% [29–35] to 35% [30–40] at the 6-month follow-up (*p* < 0.001) and to 35% [30–40] at the 12-month follow-up (*p* < 0.001), with a statistically significant improvement in both groups and no differences between AF and non-AF patients (Table 2).

The final median LVEF was 40% [35–45], with similar values in both groups. The patients without AF had higher prevalence of an LVEF increase ≥ 10% (68.4% vs. 56.1%, *p* = 0.041), but the proportion achieving an LVEF > 35% was comparable between the groups (Table 2).

**Figure 3:**
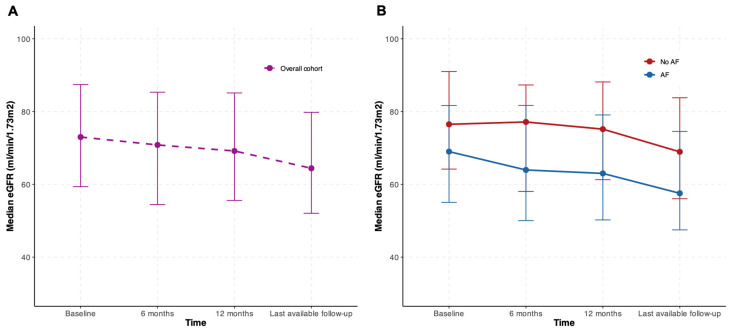
Temporal trends in changes in renal function from baseline to median follow-up. Panel (**A**), changes in eGFR (median, error bar indicating IQR) in the overall cohort. Panel (**B**), changes in eGFR (median, error bar indicating IQR) according to the presence of AF. AF, atrial fibrillation; eGFR, estimated glomerular filtration rate; IQR interquartile range.

Despite lower eGFR baseline values, the patients with AF had similar rates of renal function decline compared to the non-AF patients, with no significant differences in delta eGFR at 6 months, 12 months and last available follow-up (Table 2, Figure 3).

Consistently, WeGFR occurred in 14.6% of the patients in the overall cohort and was comparable in the two groups.

### 3.3. Risk of Adverse Events

During a median follow-up of 582 [339–1481] days, 60 (17.8%) events of the primary outcome were reported. The IR of the primary outcome was 11.57 (95% CI: 7.92–16.34) in the AF patients and almost half of this value in the non-AF ones (IR, 95% CI: 5.65, 3.76–8.17), as detailed in Table 3.

Kaplan–Meier survival curves (Figure S1) demonstrated a lower survival probability for the AF patients. The Kaplan–Meier curves for the secondary exploratory outcomes are reported in Figure S2A for all-cause death and in panel B for hHF. This result was confirmed by Cox regression analysis (Table 3), also after adjusting for covariates (aHR, 95% CI: 2.12, 1.16–3.86). Concerning the secondary exploratory outcomes, no differences were observed for the risk of all-cause death, while the AF patients showed a higher risk of hHF (aHR, 95% CI: 2.80, 1.44–5.46).

**Figure 4:**
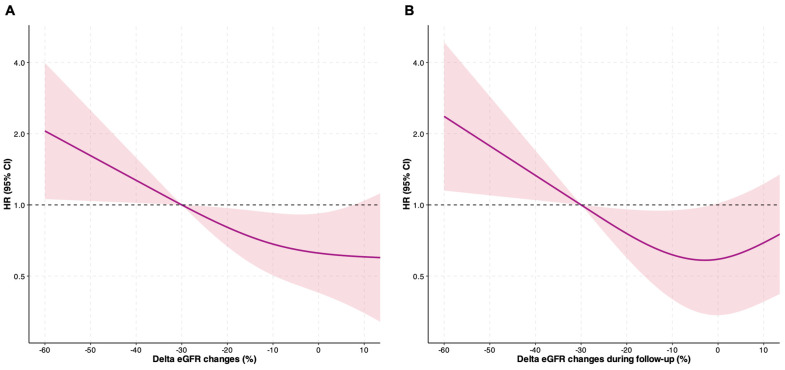
Delta changes in renal function plotted as a continuous variable and association with the primary outcome. CI, confidence interval; eGFR, estimated glomerular filtration rate; HR, hazard ratio. Panel (**A**) shows the linear relationship between delta changes in renal function and the risk of adverse events (*p* = 0.043, *p* for non-linearity = 0.329). Panel (**B**) shows the non-linear relationship between delta changes in renal function from the 6-month follow-up and the risk of adverse events (*p* = 0.005, *p* non-linearity = 0.002).

When assessing the association between delta eGFR overall changes and the risk of the primary outcome (Figure 4A), we found a statistically significant association for an increased risk of adverse events when eGFR decreased by at least 30% (*p* = 0.0443, *p* non-linearity = 0.329). Consistently, the negative delta eGFR changes from the 6-month follow-up were statistically associated with a higher risk of adverse events, with a non-linear relationship (*p* = 0.005, *p* non-linearity = 0.002). As shown in Figure 4B, the risk of the primary endpoint progressively increased for delta eGFR changes of more than −30%, while it was reduced for changes below −30% and tended to plateau when there were no changes in renal function during the follow-up.

## 4. Discussion

The principal findings of our real-world analysis in a contemporary cohort of HFrEF patients are as follow: (i) the AF patients were older, with higher RDW and NP values, yet receiving a similar GDMT, though ARNi prescription was lower in patients with AF; (ii) the LVEF improvement was more pronounced in the non-AF patients, with a higher proportion achieving a ≥10% increase, even if the absolute LVEF changes were similar between the groups; (iii) the baseline eGFR was lower in the patients with AF, but the renal function decline over time was comparable with no higher prevalence of a ≥30% decline in eGFR—a critical threshold associated with adverse outcomes in our cohort; (iv) AF in the patients with HFrEF was associated with a nearly two-fold increased risk of the composite outcome, primarily due to a three- to four-fold higher risk of hHF, with no significant differences in all-cause death.

AF patients are generally older and with a higher burden of comorbidities (i.e., hypertension, CKD, diabetes mellitus) than those without AF, which contributes to a more complex clinical profile. Among the biochemistry parameters, RDW in the AF patients seemed to be greater than in the non-AF patients, suggesting an underlying micro-inflammatory state, often associated with adverse outcomes [15,16]. Regarding the NP values, in our cohort, the different median BNP values at baseline were the expression of AF presence, within a range of values indicating biochemical stability in both groups, given the absence of different median BMI values [17].

Despite similar overall adherence to GDMT classes, we observed a lower prescription of ARNi for the AF patients, likely due to concerns about CKD, because of which ARNi is less frequently used, despite growing evidence supporting its benefit in this subgroup, and to iatrogenic hypotension, especially in older, comorbid patients [18,19]. Additional factors may include the perception, highlighted by the underrepresentation of AF patients in the RCTs, that ARNis may be less effective in this population, as well as the complexity of managing polypharmacy and comorbidities in AF patients, which may lead to more cautious prescriptions. Administrative and reimbursement restrictions, such as those within the AIFA therapeutic plan, may also contribute to limiting their use. However, increasing evidence from randomized clinical trials’ pre-specified subgroup analyses and observational studies suggests that ARNis may prevent de novo AF and slow disease progression, supporting a broader adoption of this therapy in eligible patients with AF and HFrEF [7,20].

In our study, the non-AF patients exhibited a more pronounced improvement in LVEF following optimized GDMT compared to those with AF. However, we did not observe significant differences in the absolute change in LVEF from baseline between the groups, suggesting that the AF patients still experienced a beneficial response to therapy, even if lower.

There are several possible mechanisms underlying this attenuated response [21], including the loss of atrial contraction, higher ventricular rates, and more advanced myocardial fibrosis and structural remodeling, all limiting LV filling and reverse remodeling [22]. In addition, irregular RR intervals and the chronic hemodynamic burden of AF may further impair diastolic filling and promote maladaptive remodeling. Neurohormonal activation associated with AF can contribute to diffuse myocardial fibrosis and attenuate the potential for reverse remodeling. From a therapeutic perspective, the lower prescription rates of ARNI and MRAs observed in AF patients in our cohort may partially explain the reduced likelihood of EF recovery.

These findings emphasize the need for personalized treatment approaches in AF patients with HFrEF, potentially incorporating rhythm control strategies, regardless of the symptoms, despite them being on GDMT [23,24,25]. As recently revised by Goette et al. [26], alongside HF, atrial failure is often associated with atrial cardiomyopathy, characterized by atrial fibrosis, dilatation and functional impairment and increasingly recognized as a key determinant of HF progression, independently of ventricular function [27,28]. This suggests that in addition to rate and rhythm control strategies, targeted medical interventions addressing atrial structural remodeling is required in HFrEF patients [12].

Renal function decline remains a barrier to GDMT optimization, despite the observation that medically treated HFrEF patients had a slower decline in renal function compared to patients without optimal medical therapy, especially following renin–angiotensin system inhibitor (RAASi) implementation [29]. In our analysis, while the AF patients had a persistently lower eGFR, the rate of the decline did not differ significantly from that in the non-AF patients, nor the percentage of patients experiencing a more serious eGFR reduction, in line with existing evidence [30,31]. An eGFR decline ≥ 30% was identified as a critical threshold associated with worse outcomes in our cohort, claiming for a clinical and laboratory intensified follow-up for those patients experiencing such renal function decline on GDMT [32]. The relationship between AF, renal dysfunction, and HFrEF is complex and multidirectional. AF contributes to progressive kidney disease through various mechanisms such as altered renal perfusion, neurohormonal activation and systemic inflammation. Conversely, CKD itself promotes AF development due to shared risk factors such as hypertension, volume overload and endothelial dysfunction [33]. Moreover, the lower renal function in the AF patients observed in our study may be evidence of cardiorenal syndrome, rather than a direct effect of AF on renal decline [34]. Notably, despite this consideration, in our cohort the AF patients did not exhibit a significantly greater deterioration in kidney function over time, possibly due to the optimized GDMT, including the use of RAASis and mineralocorticoid receptor antagonists, which have been shown to confer renal and metabolic protective effects in HFrEF [35].

Our findings confirm that in patients with HFrEF, AF is associated with a significantly higher risk of the composite outcome of hHF or all-cause death, mainly driven by a three- to four-fold higher risk of hHF, whereas all-cause death alone did not differ between the groups. These results reinforce prior research demonstrating the adverse prognostic impact of AF in HFrEF [36,37].

Several mechanisms may explain this increased risk. AF is associated with reduced cardiac systolic function, lower LVEF recovery and adverse cardiac remodeling, contributing to worsening HF and increased hospitalizations [38]. The irregular ventricular rhythm in AF leads to hemodynamic issues with the loss of atrial contribution to cardiac output, predisposing patients to worsening HF episodes. In addition, AF is frequently associated with poor adherence to GDMT due to concerns about renal function, hypotensive events and drug interactions, leading to clinical prescription inertia and suboptimal HFrEF therapy administrations [39]. However, in our cohort patients with and without AF were all fully prescribed RAASis, the main concerning drugs for hypotension and renal function decline, and no differences were found with respect to these two adverse events, stressing the concept of fulfilling GDMT prescription in AF patients.

The difference in mortality outcomes between AF and non-AF HFrEF patients is remarkable and documented in the literature. In our HFrEF cohort, despite higher crude rates of all-cause death in the AF patients, the association of AF with death was not robust, and its impact on survival appeared less direct, likely due to advances in HF therapies (i.e., pharmacological and remote monitoring management), a closer follow-up for such patients especially after a worsening HF episode [40] and anticoagulation therapies, mitigating the excess mortality risk traditionally associated with AF [41,42].

Thus, our findings highlight the importance of comprehensive management strategies for AF in HFrEF patients [43,44]. Given the increased risk of hHF, rhythm control strategies for AF should probably be considered in this population [45]. Despite the growing body of RCTs advocating for an early rhythm control strategy, patient selection remains a pivotal element of this therapeutic decision [46]. In many cases, the importance of optimizing GDMT for HFrEF patients before considering interventional AF strategies is overlooked. AF interventions represent only one aspect of the broader AF-HF syndrome (i.e., cardio–renal–metabolic relationships) and should be pursed in addition to the medical optimized therapy [23,47,48,49]. While further studies are needed to determine the most effective AF management strategies for real-world HFrEF populations, our findings reinforce the need to prioritize GDMT optimization for AF patients.

Some limitations should be acknowledged while interpreting our results. The retrospective and observational design of the study may have influenced our ability to explore causal associations. Furthermore, as a single-center study with a relatively small sample size and short follow-up, our results may not be fully generalizable to broader populations. Additionally, many patients were enrolled before the widespread adoption of SGLT2is, which are now a cornerstone of HF therapy. Consequently, at baseline, fewer than half of the patients received SGLT2is, which potentially limits the applicability of our findings regarding the renal function trends, given the well-established renal protective effects of SGLT2is, despite at the end of follow-up nearly 80% of the patients in our cohort receiving SGLT2i. The lack of granular data, especially concerning the AF type and burden, might have limited the possibility to identify patients with AF-related cardiomyopathy, with potentially different outcomes. All echocardiographic assessments were performed by experienced physicians in our tertiary-care center; however, minor intra- and inter-observer variability cannot be excluded. Similarly, the laboratory exams were not performed by a consistent laboratory source throughout the study, potentially introducing variability in renal function assessment. Lastly, the exclusion of patients with missing data may have introduced a selection bias, and, although multivariable analyses were conducted to adjust for potential confounders, residual confounding cannot be entirely excluded.

## 5. Conclusions

In this contemporary cohort of HFrEF patients, those with AF experienced a comparable improvement in LVEF and a similar decline in renal function (eGFR) compared to non-AF patients, underscoring the importance of optimizing GDMT, particularly through ARNi use. An eGFR decline ≥ 30% was associated with adverse outcomes, requiring closer clinical and laboratory monitoring in this high-risk population. Overall, AF was associated with a higher risk of adverse events, mainly consisting of increased HF hospitalizations, highlighting the need for prompt and comprehensive GDMT optimization in these patients.

## Figures and Tables

**Table 1:** Baseline characteristics according to the presence of atrial fibrillation.

	Overall	No AF	AF	*p* Value
N (%)	321 (100)	187 (58.3%)	134 (41.7%)	
Female sex, *n* (%)	62 (19.3)	37 (19.8)	25 (18.7)	0.913
Age (years) median [IQR]	67 [58–74]	64 [56–71]	72 [65–78]	<0.001
BMI (kg/m^2^), median [IQR]	27.68 [24.77–30.49]	28.05 [24.88–30.86]	27.17 [24.69–30.47]	0.266
NYHA class, median [IQR]	2 [2–3]	2 [2–2]	2 [2–3]	0.202
Ischemic etiology, *n* (%)	153 (47.8)	93 (49.7)	60 (45.1)	0.483
LVEF (%), median [IQR]	31 [29–35]	30 [28–35]	32 [30–35]	0.011
Hypertension, *n* (%)	215 (67.0)	124 (66.3)	91 (67.9)	0.857
Systolic BP (mmHg), median [IQR]	120 [110–130]	120 [110–130]	120 [110–130]	0.204
Diastolic BP (mmHg), median [IQR]	75 [70–80]	75 [70–80]	75 [70–80]	0.419
Diabetes, *n* (%)	69 (21.5)	46 (24.6)	23 (17.2)	0.144
Dyslipidemia, *n* (%)	228 (71.0)	137 (73.3)	91 (67.9)	0.359
Smoking, *n* (%)				0.083
Never	159 (49.7)	86 (46.0)	73 (54.9)	
Former	114 (35.6)	67 (35.8)	47 (35.3)	
Current	47 (14.7)	34 (18.2)	13 (9.8)	
PAD, *n* (%)	20 (7.5)	9 (5.6)	11 (10.4)	0.230
Carotid artery disease, *n* (%)	23 (7.2)	10 (5.3)	13 (9.7)	0.203
TE events, *n* (%)	19 (5.9)	11 (5.9)	8 (6.0)	1.000
Previous ACS, *n* (%)	105 (32.7)	66 (35.3)	39 (29.1)	0.296
Device, *n* (%)				0.285
No	94 (29.7)	52 (28.0)	42 (32.3)	
ICD	153 (48.4)	93 (50.0)	60 (46.2)	
CRT-D	62 (19.6)	39 (21.0)	23 (17.7)	
CRT-P	7 (2.2)	2 (1.1)	5 (3.8)	
CKD, *n* (%)	80 (26.7)	32 (18.5)	48 (37.8)	<0.001
eGFR (mL/min/1.73 m^2^), median [IQR]	73.00 [59.38–87.43]	76.50 [64.20–91.00]	69.00 [55.05–81.65]	<0.001
Kalemia (mEq/L), median [IQR]	4.30 [3.90–4.60]	4.30 [4.00–4.60]	4.40 [3.90–4.70]	0.495
Hb (g/dL), median [IQR]	13.80 [12.90–15.10]	13.90 [13.05–15.10]	13.50 [12.60–15.15]	0.239
RDW (%), median [IQR]	14.10 [13.40–15.40]	13.80 [13.20–14.60]	14.80 [13.62–15.75]	<0.001
BNP (ng/L), median [IQR]	291 [135–570]	192 [96–453]	366 [227–642]	0.004
Treatments				
ARNi, *n* (%)	241 (75.1)	150 (80.2)	91 (67.9)	0.017
ACEi, *n* (%)	26 (8.1)	14 (7.5)	12 (8.9)	0.427
ARB, *n* (%)	42 (13.1)	22 (11.8)	20 (14.9)	0.473
MRA, *n* (%)	267 (83.2)	153 (81.8)	114 (85.1)	0.537
Betablocker, *n* (%)	311 (96.9)	180 (96.3)	131 (97.8)	0.660
SLGT2i, *n* (%)	124 (38.6)	64 (34.2)	60 (44.8)	0.072
Diuretics, *n* (%)	272 (85.0)	154 (82.8)	118 (88.1)	0.253
OAC, *n* (%)	158 (49.7)	31 (16.8)	127 (94.8)	<0.001
SAPT, *n* (%)	114 (35.7)	94 (50.8)	20 (14.9)	<0.001
Long DAPT, *n* (%)	11 (3.5)	10 (5.5)	1 (0.8)	0.052

ACEi, angiotensin-converting enzyme inhibitor; ACS, acute coronary syndrome; AF, atrial fibrillation; ARB, angiotensin receptor blocker; ARNi, angiotensin receptor–neprilysin inhibitor; BMI, body mass index; BNP, brain natriuretic peptide; BP, blood pressure; CKD, chronic kidney disease; CRT-D, cardiac resynchronization therapy defibrillator; CRT-P, cardiac resynchronization therapy pacemaker; DAPT, dual antiplatelet therapy; eGFR, estimated glomerular filtration rate; ICD, implantable cardioverter defibrillator; IQR, interquartile range; LVEF, left ventricular ejection fraction; MRA, mineralocorticoid receptor antagonist; NYHA, New York Heart Association; OAC, oral anticoagulant; RDW, red cell distribution width; SAPT, single antiplatelet therapy; SGLT2i, sodium–glucose transport protein 2 inhibitor; TE, thromboembolic.

**Table 2:** Follow-up values and changes in left ventricular ejection fraction and renal function at 6-, 12-month and last available follow-ups.

	Overall	No AF	AF	*p* Value
Estimated glomerular filtration rate				
eGFR (mL/min/1.73 m^2^), median [IQR]	73.00 [59.38–87.43]	76.50 [64.20–91.00]	69.00 [55.05–81.65]	<0.001
eGFR 6-month FU, median [IQR]	70.83 [54.44–85.32]	77.15 [58.06–87.31]	63.96 [50.04–81.70]	0.002
eGFR 12-month FU, median [IQR]	69.17 [55.58–85.12]	75.17 [61.28–88.14]	63.01 [50.23–79.07]	0.001
eGFR last available FU, median [IQR]	64.42 [52.05–79.79]	68.94 [56.05–83.79]	57.55 [47.49–74.53]	<0.001
Delta eGFR 6-month, median [IQR]	−2.66 [−9.99–4.06]	−2.51 [−9.75–4.79]	−3.21 [−10.34–2.61]	0.708
Delta eGFR 12-month, median [IQR]	−2.86 [−12.17–3.15]	−2.72 [−12.28–2.63]	−3.64 [−11.95–4.11]	0.944
Delta eGFR overall, median [IQR]	−10.29 [−20.82–1.56]	−10.43 [−19.44–(−0.05)]	−9.88 [-26.81–3.17]	0.945
WeGFR, *n* (%)	42 (14.6)	20 (11.9)	22 (18.3)	0.176
Left ventricular ejection fraction				
LVEF (%), median [IQR]	31 [29–35]	30 [28–35]	32 [30–35]	0.011
LVEF 6-month FU, median [IQR]	35 [30–40]	35 [30–40]	35 [30–40]	0.858
LVEF 12-month FU, median [IQR]	35 [30–40]	35 [30–40]	35 [30–43]	0.752
LVEF last available FU, median [IQR]	40 [35–45]	40 [35–45]	39 [34–47]	0.759
Delta LVEF 6-month, median [IQR]	0 [0–8]	0 [0–9]	0 [0–6]	0.103
Delta LVEF 12-month, median [IQR]	2 [0–9]	3 [0–10]	1 [0–7]	0.155
Delta LVEF overall, median [IQR]	7 [0–15]	8 [2–15]	5 [0–15]	0.206
LVEF ≥ 10% from baseline, *n* (%)	190 (63.3)	121 (68.4)	69 (56.1)	0.041
ImpLVEF, *n* (%)	187 (62.3)	114 (64.4)	73 (59.3)	0.443

AF, atrial fibrillation; eGFR, estimated glomerular filtration rate; FU, follow-up; ImpLVEF, improved left ventricular ejection fraction; IQR, interquartile range; LVEF, left ventricular ejection fraction; WeGFR, worsened estimated glomerular filtration rate.

**Table 3:** Event count, incidence rates and Cox regression analysis for the risk of the primary endpoint according to the presence of atrial fibrillation.

	Event Count	IR Per 100 Person-Year (95% CI)	uHR (95% CI)	aHR * (95% CI)	aHR° (95% CI)
Primary outcome	60 (18.7)				
No AF	28 (15.0)	5.65 (3.76–8.17)	Ref.	Ref.	Ref.
AF	32 (23.9) 0.061	11.57 (7.92–16.34)	2.03 (1.22–3.38)	1.96 (1.07–3.58)	2.12 (1.16–3.86)
All-cause death	26 (8.1)				
No AF	13 (7.0)	2.46 (1.31–4.20)	Ref.	Ref.	Ref.
AF	13 (9.7) 0.495	4.01 (2.13–6.85)	1.69 (0.78–3.65)	0.87 (0.35–2.12)	1.06 (0.42–2.67)
hHF	46 (14.3)				
No AF	19 (10.2)	3.83 (2.31–5.98)	Ref.	Ref.	Ref.
AF	27 (20.1) 0.018	9.75 (6.43–14.19)	2.46 (1.37–4.44)	2.66 (1.35–5.26)	2.80 (1.44–5.46)

AF, atrial fibrillation; aHR, adjusted hazard ratio; CI, confidence interval; hHF, hospitalization for heart failure; IR, incidence rate; LVEF, left ventricular ejection fraction; uHR, unadjusted hazard ratio. * Adjusted for age, sex, LVEF, hypertension, eGFR. °Adjusted for age, sex, ischemic etiology, LVEF, hypertension, diabetes, eGFR.

## Data Availability

The data underlying this article will be shared upon reasonable request to the corresponding author.

## Supplementary Materials

The following supporting information can be downloaded at: https://www.mdpi.com/article/10.3390/jcdd12090328/s1, Figure S1: Flow-chart of the study; Figure S2: Kaplan–Meier curves for the secondary exploratory outcomes.

## Abbreviations

The following abbreviations are used in this manuscript:

AF	Atrial fibrillation
ARNi	Angiotensin receptor–neprilysin inhibitor
CKD	Chronic kidney disease
eGFR	Estimated glomerular filtration rate
GDMT	Guideline-directed medical therapy
HFrEF	Heart failure with reduced ejection fraction
hHF	Hospitalization for heart failure
LVEF	Left ventricular ejection fraction
NYHA	New York Heart Association
RAASis	Renin–angiotensin system inhibitors
RDW	Red cell distribution width
SGLT2is	Sodium–glucose transport protein 2 inhibitors
