# Supplementary material for: Real-World Use of ARNI Within GDMT in HFrEF Patients with and Without Atrial Fibrillation: A Retrospective Analysis of Cardiac and Renal Functions and Clinical Outcomes
Source: J Cardiovasc Dev Dis. 2025 Aug 26;12(9):328. doi: 10.3390/jcdd12090328 (PMC12470668; doi:10.3390/jcdd12090328)
Supplement: Supplementary file 1 [file jcdd-12-00328-s001.zip › jcdd-3791546-supplementary.pdf]

Figure S1. Kaplan-Meier curves for the primary outcome according to the presence of atrial fibrillation.

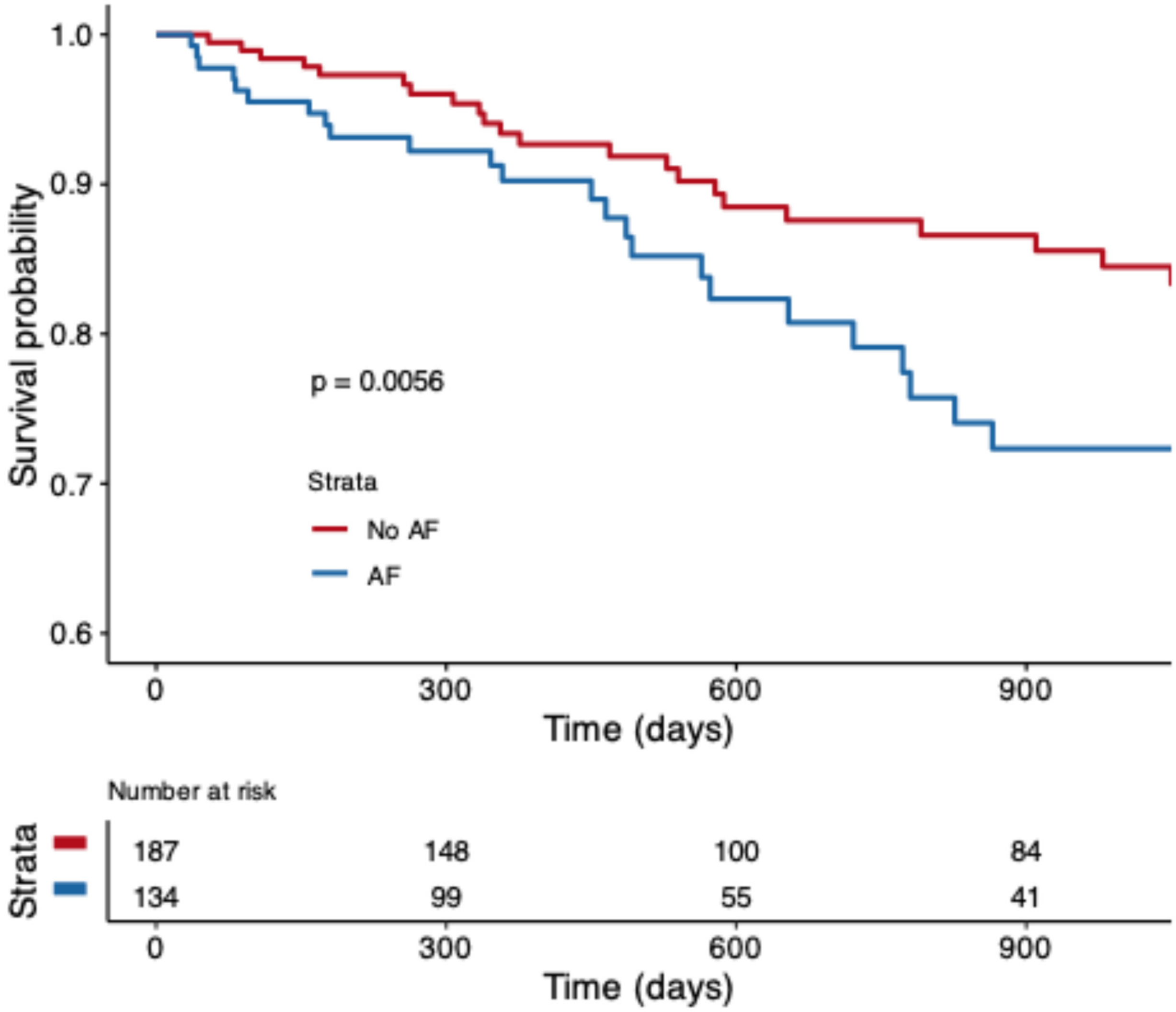

Legend: AF, atrial fibrillation.

Figure S2. Kaplan Meier curves for the secondary exploratory outcomes.

Panel A)

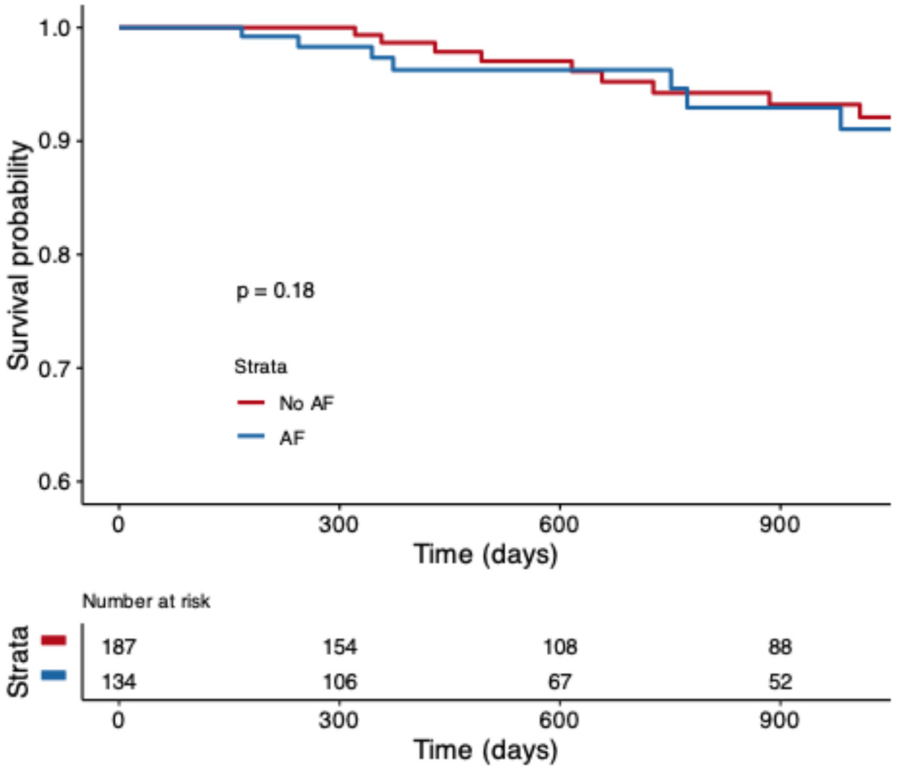

Panel B)

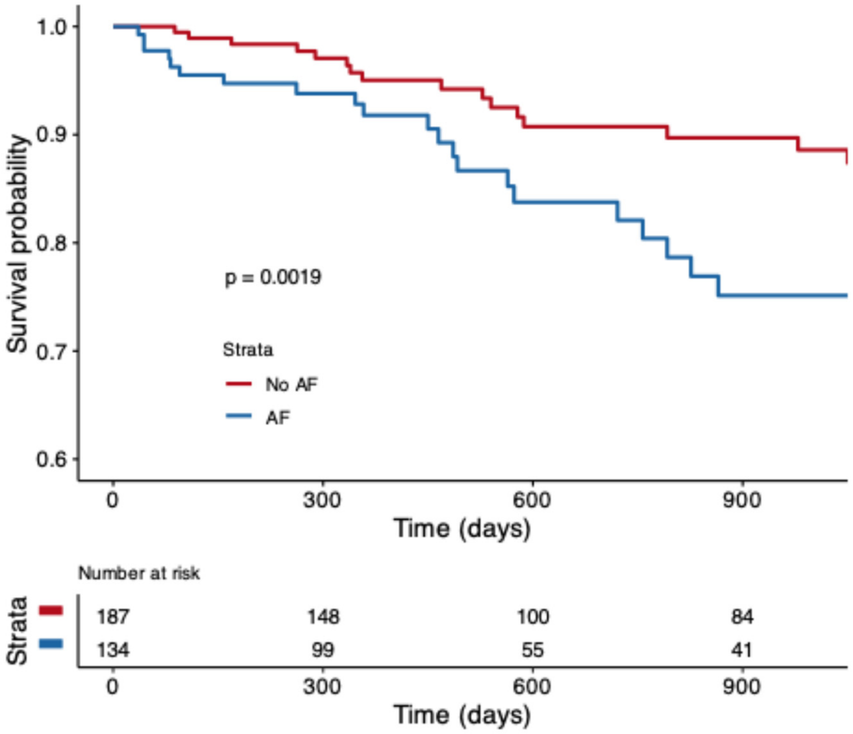

Legend: AF, atrial fibrillation; hHF, hospitalization for heart failure.
Panel A) all-cause death
Panel B) hHF
